# Differential DNA accessibility to polymerase enables 30-minute phenotypic β-lactam antibiotic susceptibility testing of carbapenem-resistant Enterobacteriaceae

**DOI:** 10.1371/journal.pbio.3000652

**Published:** 2020-03-19

**Authors:** Nathan G. Schoepp, Eric J. Liaw, Alexander Winnett, Emily S. Savela, Omai B. Garner, Rustem F. Ismagilov

**Affiliations:** 1 Division of Chemistry and Chemical Engineering, California Institute of Technology, Pasadena, California, United States of America; 2 Division of Biology and Biological Engineering, California Institute of Technology, Pasadena, California, United States of America; 3 Department of Pathology and Laboratory Medicine, UCLA, Los Angeles, California, United States of America; Universitat zu Koln, GERMANY

## Abstract

The rise in carbapenem-resistant Enterobacteriaceae (CRE) infections has created a global health emergency, underlining the critical need to develop faster diagnostics to treat swiftly and correctly. Although rapid pathogen-identification (ID) tests are being developed, gold-standard antibiotic susceptibility testing (AST) remains unacceptably slow (1–2 d), and innovative approaches for rapid phenotypic ASTs for CREs are urgently needed. Motivated by this need, in this manuscript we tested the hypothesis that upon treatment with β-lactam antibiotics, susceptible Enterobacteriaceae isolates would become sufficiently permeabilized, making some of their DNA accessible to added polymerase and primers. Further, we hypothesized that this accessible DNA would be detectable directly by isothermal amplification methods that do not fully lyse bacterial cells. We build on these results to develop the polymerase-accessibility AST (pol-aAST), a new phenotypic approach for β-lactams, the major antibiotic class for gram-negative infections. We test isolates of the 3 causative pathogens of CRE infections using ceftriaxone (CRO), ertapenem (ETP), and meropenem (MEM) and demonstrate agreement with gold-standard AST. Importantly, pol-aAST correctly categorized resistant isolates that are undetectable by current genotypic methods (negative for β-lactamase genes or lacking predictive genotypes). We also test contrived and clinical urine samples. We show that the pol-aAST can be performed in 30 min sample-to-answer using contrived urine samples and has the potential to be performed directly on clinical urine specimens.

## Abstract 2

By directly linking beta-lactam-induced cell wall damage to a rapid DNA measurement, this study introduces a new concept for creating phenotypic antibiotic-susceptibility tests. The concept was validated with carbapenem-resistant Enterobacteriaceae, considered one of the top three most-urgent antibiotic resistant threats by the Centers for Disease Control.

## Introduction

The evolution and global spread of carbapenem-resistant Enterobacteriaceae (CRE) threatens to disrupt modern healthcare systems, which rely heavily on β-lactams (especially carbapenems, the last-resort treatments) to control bacterial infections [1–3]. Mortality rates for CRE infections are as high as 30%–49% [4–6], and thus the global emergence and spread of CRE infections represents a public health emergency [7–9]. The Centers for Disease Control and Prevention (CDC) places CRE in its highest (“urgent”) category of antimicrobial-resistant pathogen threats [8,10], and the World Health Organization (WHO) labels CRE as a critical-priority pathogen [7]. *Escherichia coli*, *Klebsiella pneumoniae*, and *Enterobacter* spp. compose the majority of CRE infections and are the most commonly monitored Enterobacteriaceae [8,11–13].

To halt the further spread of CRE, patients need to be treated swiftly and correctly at the point of care (POC); however, there is no fast and general method for determining antibiotic susceptibility [14–16]. The current clinical workflow for treatment of bacterial infections consists of an identification (ID) step followed by an antibiotic susceptibility test (AST). Although progress is being made to develop faster ID tests [17–19] and a rapid 20-min ID test is on the horizon [20–22], the gold-standard for AST remains a culture-based workflow using broth or agar dilution that requires 1 to 2 d and is thus far too slow [23,24]. Because AST results are so delayed, healthcare providers usually treat empirically, leading to inappropriate prescriptions and even life-threatening outcomes [25], as well as the further spread of resistance. To improve treatment and promote antibiotic stewardship, healthcare providers need a rapid phenotypic AST [26–29].

ASTs are either genotypic or phenotypic. Genotypic tests predict resistance by measuring the presence of genes known to be involved in resistance. Genotypic tests can be fast [30] but often have limited clinical utility because they target defined mechanisms of resistance. For example, rapid genotypic methods to detect gram-negative β-lactamase genes have been developed [31–34], but these tests only detect one of the many known β-lactamase classes and still require 30 to 40 min (estimated from described methods). Similarly, the commercial Cepheid Xpert Carba-R assay (Cepheid, Sunnyvale, CA), which detects 5 β-lactamase gene families, was shown to detect 50% of resistant isolates and took 88 min [35]. Moreover, although Carba-R is Food and Drug Administration (FDA) approved, its utility in treatment scenarios is limited (i.e., negative results are not actionable), so when prescribing antibiotics, it must be used in conjunction with a phenotypic AST [36,37]. Rapid methods for measuring the activity of specific β-lactamases also exist [38–42]. However, these tests only detect one mechanism of resistance, and sample-to-answer times have not been reported.

Phenotypic ASTs are ideal because they determine susceptibility directly by exposing the sample to antibiotics and measuring the target organism’s response. The gold-standard AST (broth microdilution [23,24]) is a phenotypic test. Most phenotypic tests require the growth of viable organisms isolated from patient samples, a process that requires days and is thus too slow for the POC. Innovative, faster phenotypic tests for β-lactams were developed based on in situ nucleic-acid staining or fluorescence measurements [43–45], flow cytometry [46], microscopy [47–49], optical density [50,51], and mass spectrometry [52]. However, the majority of the currently proposed methods still require 60- to 180-min antibiotic-exposure steps in addition to the time needed to perform the assay, and no method has emerged that achieves short (approximately 15 min) antibiotic exposure and short (approximately 15 min) assay time but does not require excessively complex or delicate instrumentation so the method can be deployed at the POC.

Rapid phenotypic methods based on quantification of nucleic acids (NAs) have shown great promise for a rapid POC AST due to the speed, specificity, and robustness of NA detection [53–58]. There is an additional advantage to using NA quantification as a readout of the bacterial response to antibiotic: because rapid pathogen ID from clinical samples is commonly performed via NA analysis, it would likely be easier to integrate an NA-based phenotypic AST into a combined ID/AST workflow performed from the same clinical sample. Additionally, the use of NA-based methods provides molecular specificity towards the target pathogen, which is important in clinical samples that can contain multiple organisms. For antibiotics that directly or indirectly impact NA replication on short timescales, we have demonstrated that the quantification of DNA [59,60] or RNA [61] can be used to rapidly (30 min) and reliably determine susceptibility to nitrofurantoin and ciprofloxacin. Subsequent efforts have targeted the β-lactam class (the most widely prescribed class of antibiotic [1,2]) using these methods [62]. However, because β-lactams do not directly impact NA replication on short timescales, this direct translation of the existing NA-based technique required a 2-h antibiotic exposure, which is not sufficiently rapid for the POC. For a POC AST to impact management of CRE infections, it must (i) determine susceptibility to β-lactams, including carbapenems; (ii) be rapid (<30-min sample-to-answer) [63,64]; and (iii) be phenotypic [26,27]. As discussed subsequently, rapid pathogen ID technologies are becoming available, and therefore pathogen ID is not the focus of this work.

Here, we hypothesized that a new NA-based approach could be used to develop a rapid phenotypic AST for multiple β-lactams. We hypothesized that upon treatment with β-lactam antibiotics, susceptible Enterobacteriaceae isolates would become sufficiently permeabilized so some of their DNA would become accessible to added polymerase and primers. Further, we hypothesized that this accessible DNA would be detectable directly by isothermal amplification methods that do not fully lyse bacterial cells. To differentiate between resistant and susceptible organisms, rather than measuring how total NA concentration is impacted by antibiotic exposure (as in previous NA-based ASTs), we hypothesized that we could measure the accessibility of NAs to polymerase following a short antibiotic exposure. Here, we test these hypotheses and use them to design a new AST method, termed polymerase-accessibility AST (pol-aAST). To validate the method, we performed 82 ASTs using clinical isolates of 3 major CRE pathogens exposed to each of 3 commonly prescribed β-lactams for gram-negative infections: ceftriaxone (CRO), ertapenem (ETP), and meropenem (MEM). To further demonstrate that this method has potential to be used clinically in POC-relevant timescales, we (i) performed timed sample-to-answer experiments using contrived urine samples to ensure that the whole assay can be performed in <30 min, and (ii) we performed a pilot study on clinical urine samples from patients with urinary tract infections (UTIs).

## Results

**Fig 1:**
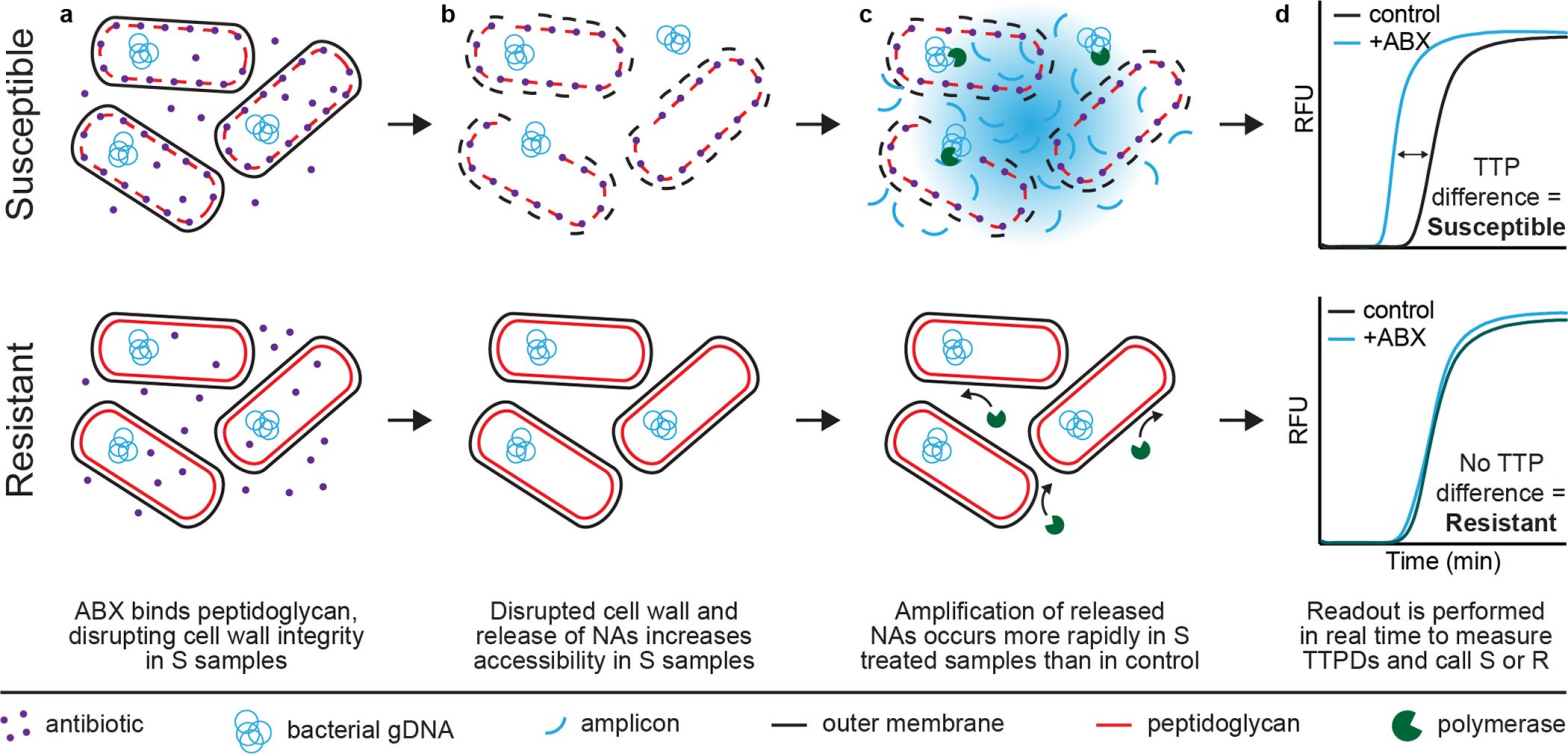
Overview of the pol-aAST shown for susceptible and resistant samples exposed to β-lactams. (a) Treated aliquots are exposed to a β-lactam. In susceptible samples, β-lactams compromise cell wall integrity. (b) NAs are released from compromised cells, increasing NA accessibility to polymerase. (c) Released NAs in the susceptible treated aliquot amplify faster than NAs from intact cells in the control aliquot, resulting in a difference in TTP. No difference in amplification between control and treated aliquots is observed in resistant samples. (d) TTPD between control and treated aliquots is used to assess susceptibility. ABX, antibiotic; AST, antibiotic susceptibility testing; gDNA, genomic DNA; NA, nucleic acid; pol-aAST, polymerase-accessibility AST; R, resistant; RFU, relative fluorescent units; S, susceptible; TTP, time-to-positive; TTPD, time-to-positive difference.

The pol-aAST relies on differential accessibility of NAs to polymerases as a result of antibiotic exposure. In this manuscript, we define differential accessibility to polymerase as a difference in the measured rate of amplification between control and antibiotic-treated samples. In the first step of pol-aAST, a single sample is split into control and treated aliquots of equal volume, and the treated aliquot is exposed to a β-lactam. Antibiotic exposure is a critical step in any phenotypic AST because phenotypic tests measure the response of bacteria to antibiotics. If the bacteria in the sample are resistant, we hypothesized that no differences in NA amplification would be observed between control and treated aliquots. If the bacteria are susceptible, we hypothesized that antibiotic treatment would lead to a compromised peptidoglycan cell wall (Fig 1A) and partial release of NAs (Fig 1B). We hypothesized that both the compromised cell wall and partial release of NAs would increase the accessibility of NAs to polymerase in a treated antibiotic-susceptible aliquot. In the second step of pol-aAST, control and treated aliquots are exposed to polymerase in amplification conditions (Fig 1C), and the rate of amplification is measured.

To successfully differentiate susceptible and resistant samples, ideal amplification conditions must (i) not fully lyse cells, (ii) enhance alterations (damage) to the cell wall caused by exposure to β-lactams, and (iii) increase NA release only from antibiotic-damaged cells. The rate of amplification is dependent on the concentration of polymerase-accessible NA. In susceptible samples, more NAs are released in the treated aliquot, leading to faster amplification in susceptible treated aliquots (Fig 1D) relative to the controls. Resistant samples are not affected by the antibiotic, so control and treated aliquots have similar NA release and time-to-positive (TTP). In these samples, the low concentration of naturally occurring extracellular DNA is ultimately amplified, but at a slower rate. Amplification rate in an isothermal amplification reaction is quantified by measuring the TTP, the time it takes the reaction fluorescence to reach a predetermined threshold. We found that using pol-aAST, isolates susceptible to the β-lactam being tested show increased accessibility of NAs to polymerase, manifesting in an earlier TTP relative to the control. The TTPs of any two samples, such as the control and treated aliquots, can be compared to generate a TTP difference (TTPD) value, which can then be used to determine susceptibility by comparing to a susceptibility threshold. Here, we used the DNA polymerase *Bst* 3.0 (New England Biolabs [NEB], Ipswitch, MA) under loop-mediated isothermal amplification (LAMP) conditions.

**Fig 2:**
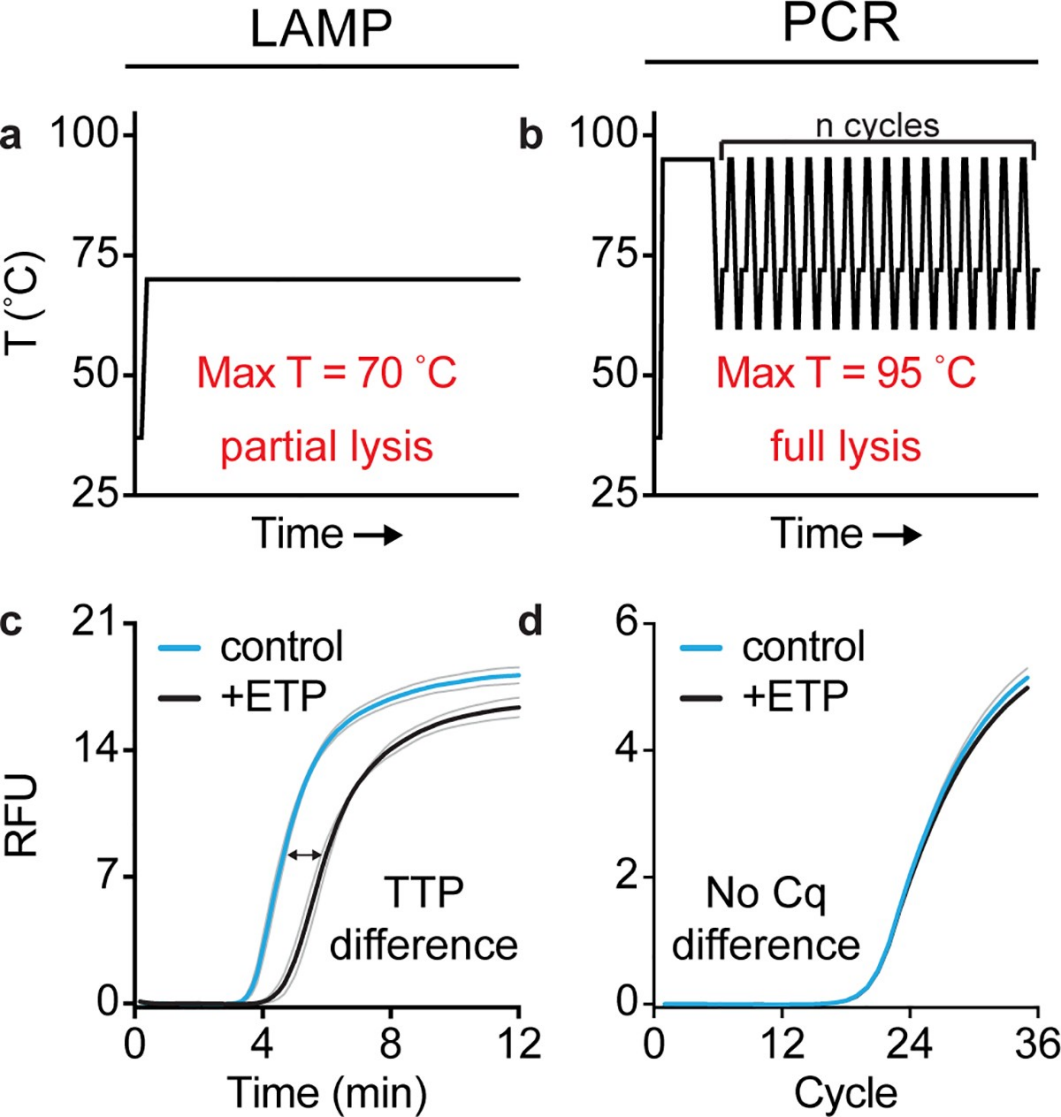
The pol-aAST requires non-lytic amplification conditions. (a–b) Thermal profiles of LAMP and PCR. (c–d) LAMP and PCR amplification curves for a susceptible *E*. *coli* isolate exposed to ETP for 15 min. Blue and black lines are the average of triplicate samples. Grey lines represent standard deviation of triplicates. A difference in TTP for control and treated aliquots is observed for susceptible isolates when quantifying NAs using LAMP, but not PCR. Raw data are provided in S5 Table. AST, antibiotic susceptibility test/testing; Cq, quantitation cycle; ETP, ertapenem; LAMP, loop-mediated isothermal amplification; NA, nucleic acid; PCR, polymerase chain reaction; pol-aAST, polymerase-accessibility AST; RFU, relative fluorescent units; TTP, time-to-positive.

We hypothesized that the chemical environment in which amplification occurs would significantly impact the result of pol-aAST and that—for pol-aAST to differentiate susceptible and resistant samples—amplification conditions should not be fully lysing. To test this, we performed pol-aAST using LAMP, as well as quantitative PCR (qPCR) (Fig 2). LAMP is performed at a single temperature (70°C), which we hypothesized would not be fully lysing, whereas qPCR is a thermocycled amplification technique reaching a maximum temperature of 95°C, which we hypothesized would be fully lysing. Indeed, we observed that pol-aAST was successful in differentiating susceptible and resistant isolates when performed using LAMP, but not when performed using qPCR (Fig 2). We tested qPCR with a total of 2 susceptible and 2 resistant isolates, none of which showed a statistically significant difference in quantitation cycle (Cq) between control and treated samples. When using LAMP, detectable differences were observed between control and treated aliquots when using isolates susceptible to the target β-lactam (TTPD = 1.02 min). Additionally, the presence of cells not lysed during LAMP is evidenced by the shorter TTPs seen when an aliquot of the same sample is lysed using an extraction buffer prior to performing LAMP (explained in more detail subsequently). These differences confirm that choice of amplification chemistry is critical to the success of pol-aAST and are consistent with previous work evaluating thermal lysis [65].

**Fig 3:**
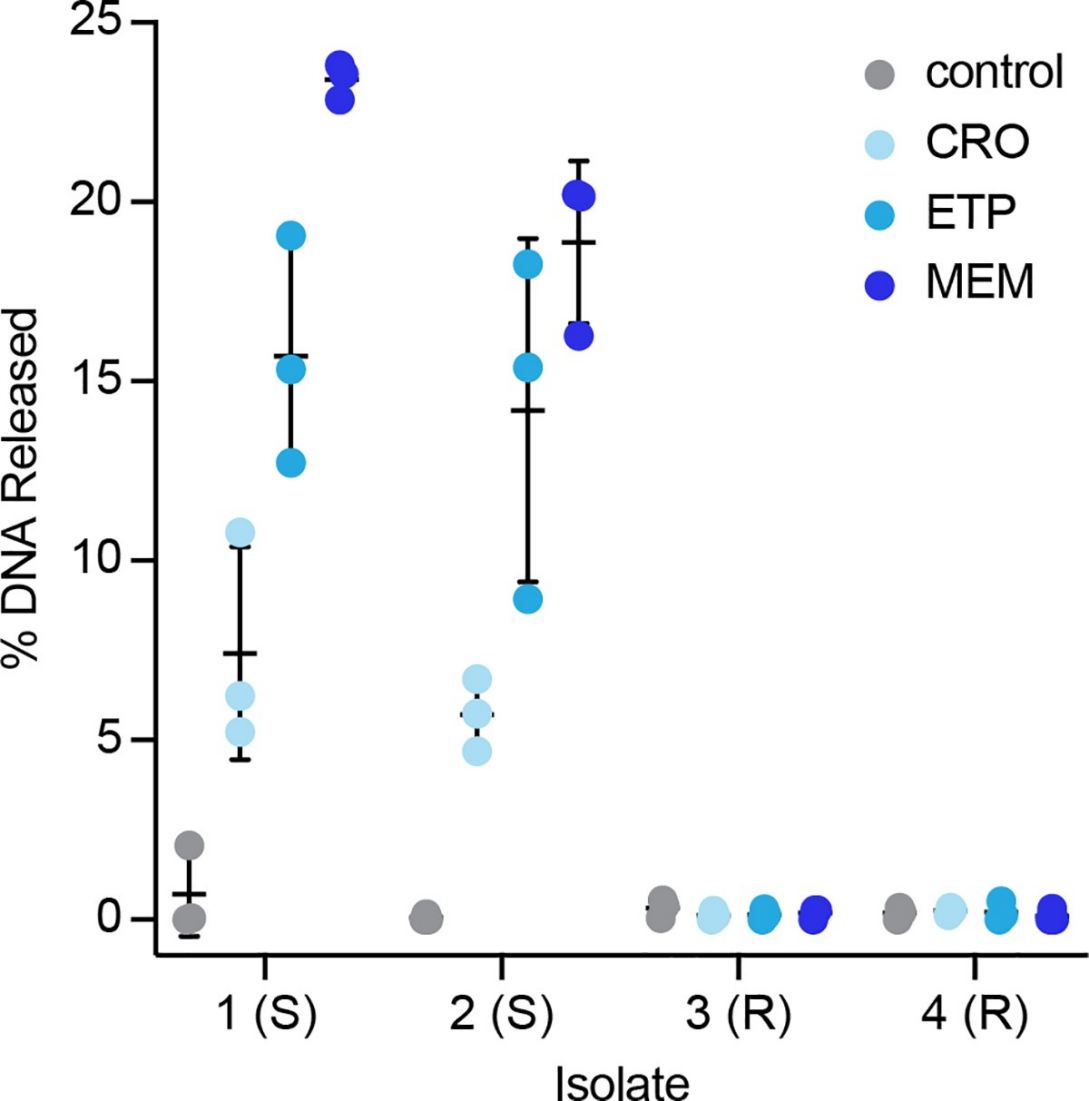
Percentage of DNA released following antibiotic exposure. Two susceptible and two resistant *E*. *coli* isolates were exposed to no antibiotic (control), CRO, ETP, or MEM for 15 min before filtering to separate intact cells from extracellular DNA. Experiments were performed in triplicate for all isolate/antibiotic combinations. Each point represents a single experiment; lines represent the average and standard deviation of replicate experiments. Raw data are provided in S6 Table. ABX, antibiotic; CRO, ceftriaxone; ETP, ertapenem; MEM, meropenem; R, resistant; S, susceptible.

To investigate the mechanism of pol-aAST, we performed experiments to separate free NAs from NAs contained within structurally intact cells or associated with cell debris. Susceptible and resistant clinical isolates were exposed to one or more β-lactams in parallel for 15 min, then filtered through 0.2 μM filters to remove cells from free NAs. NAs in the sample and eluate were then quantified using droplet digital PCR (ddPCR). We observed that following exposure to β-lactams, susceptible isolates treated with β-lactams released a significantly larger percentage of DNA than resistant samples (Fig 3). The amount of DNA released depended on the antibiotic being tested. Exposure to MEM resulted in an average of 21% of DNA being released from susceptible isolates, with a slightly smaller average percent (15%) released as a result of exposure to ETP. Interestingly, susceptible samples only released an average of 6% of DNA when exposed to CRO, demonstrating that NA release is dependent on choice of antibiotic and not, e.g., a universal stress response. These results also demonstrate that the magnitude of the effect of a β-lactam on cell wall integrity can be measured and is different depending on the antibiotic used, even on short exposure timescales.

To validate the pol-aAST method, we first performed 82 ASTs using 12 clinical isolates of *E*. *coli*, 8 clinical isolates of *K*. *pneumoniae*, 9 clinical isolates of 2 species of *Enterobacter* (*E*. *aerogenes* and *E*. *cloacae*, collectively “*Ebs*”), and the β-lactams CRO, ETP, and MEM. The set included isolates from each genus that were susceptible and isolates that were resistant to each of the 3 antibiotics. In addition to isolates obtained from the UCLA Clinical Microbiology Laboratory (CML; see Methods), those tested included *E*. *coli* and *K*. *pneumoniae* isolates from the CDC Enterobacteriaceae Carbapenem Breakpoint panel [66], as well as all available *Enterobacter* spp. isolates from the same panel. All samples were amplified using quantitative LAMP, and categorical agreement was compared to gold-standard broth microdilution AST. Two approaches for determining susceptibility were investigated in all pol-aASTs performed.

**Fig 4:**
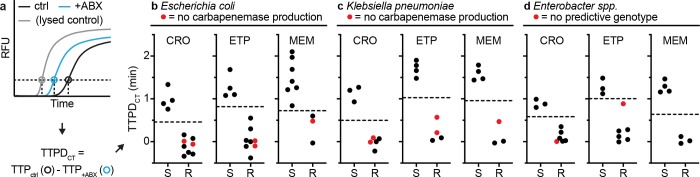
Validation of the pol-aAST method using control and antibiotic-treated aliquots. (a) Example calculation of TTPD between control and treated aliquots (TTPD_CT_). The TTP (in minutes) of the control and treated aliquots are used to calculate TTPD_CT_. (b–d) The pol-aAST results using *E*. *coli* (b), *K*. *pneumoniae* (c), and *Enterobacter* spp. (d) isolates exposed to CRO, ETP, and MEM for 15 min. Red points represent isolates with either no detectable carbapenemase genes (*Ec* and *Kp* isolates) according to a published genotypic assay [67] and commercial assay [68] or no predictive genotype (*Ebs* isolates) according to the whole genome sequencing by the CDC [66]. S/R thresholds (dashed lines) were set halfway between the lowest susceptible and the highest resistant TTPD_CT_ values. Raw data are provided in S3 Table. +ABX, antibiotic-treated; AST, antibiotic susceptibility testing; CDC, Centers for Disease Control and Prevention; CRO, ceftriaxone; CT, control to treated; ctrl, control; *Ebs*, *E*. *aerogenes* and *E*. *cloacae* collectively; *Ec*, *E*. *coli*; ETP, ertapenem; *Kp*, *K*. *pneumoniae*; MEM, meropenem; pol-aAST, polymerase-accessibility AST; R, resistant; RFU, relative fluorescent units; S, susceptible; TTP, time-to-positive; TTPD, time-to-positive difference; TTPD_CT_, TTPD control to treated.

The first approach we investigated was to compare the difference in TTP values of the control and treated aliquots in each pol-aAST. This difference was defined as TTPD control to treated (TTPD_CT_) (Fig 4A). Using the TTPD_CT_ method, we obtained 100% categorical agreement with gold-standard AST for all antibiotics tested with *E*. *coli* (Fig 4B), *K*. *pneumoniae* (Fig 4C), and *Ebs* (Fig 4D) isolates, even with resistant isolates for which the genotypic tests fail to correctly predict the resistance phenotype (red points in Fig 4). The values of TTPD_CT_ were well-separated between susceptible and resistant isolates in all CRE-antibiotic combinations. Note that the threshold values separating TTPD_CT_ of susceptible and resistant isolates depend on the antibiotic used (e.g., CRO gives a smaller response and therefore requires a lower threshold), as well as the pathogen tested (e.g., *K*. *pneumoniae* gives stronger response and requires a higher threshold). The area under the curve (AUC) of the receiver operating characteristic (ROC) curve was 1.00 for all isolates and antibiotics tested. There were no errors relative to gold-standard AST when determining susceptibility by TTPD_CT_.

**Fig 5:**
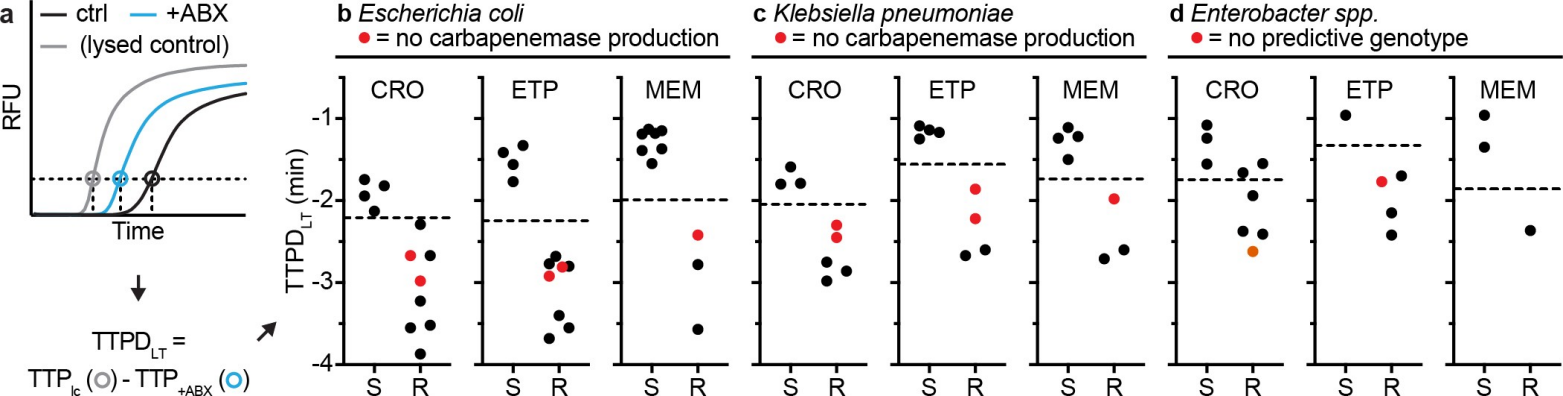
Validation of the pol-aAST method using lysed control and antibiotic-treated aliquots. (a) Example calculation of TTPD between the lysed control and antibiotic-treated aliquots (TTPD_LT_). The TTP (in minutes) in the lysed control and antibiotic-treated aliquots are used to calculate TTPD_LT_. (b–d) The pol-aAST results using *E*. *coli* (b), *K*. *pneumoniae* (c), and *Enterobacter* spp. (d) isolates exposed to CRO, ETP, and MEM for 15 min. Red points represent isolates with either no detectable carbapenemase genes (*Ec* and *Kp* isolates) according to a published genotypic assay [67] and commercial assay [68], or no predictive genotype (*Ebs* isolates) according to the CDC [66]. S/R thresholds (dashed lines) were set halfway between the lowest susceptible and the highest resistant TTPD_LT_ values except in the case of *Enterobacter* spp. treated with CRO (see text). Raw data are provided in S3 Table. +ABX, antibiotic-treated; AST, antibiotic susceptibility testing; CDC, Centers for Disease Control and Prevention; ctrl, control; CRO, ceftriaxone; *Ebs*, *E*. *aerogenes* and *E*. *cloacae* collectively; *Ec*, *E*. *coli*; ETP, ertapenem; *Kp*, *K*. *pneumoniae*; lc, lysed control; MEM, meropenem; pol-aAST, polymerase-accessibility AST; R, resistant; RFU, relative fluorescent units; S, susceptible; TTP, time-to-positive; TTPD, time-to-positive difference; TTPD_LT_, TTPD lysed-control to treated.

The second approach we investigated was to compare the difference in TTP values of a fully lysed aliquot and the antibiotic-treated aliquot in each pol-aAST. The fully lysed aliquot was created by extracting NA from the antibiotic-treated sample using a single-step, LAMP-compatible extraction buffer. This difference was defined as TTPD lysed-control to treated (TTPD_LT_) (Fig 5A). It is important to note that TTPD_LT_ only requires an antibiotic-treated sample during the exposure step (the method does not require the use of a no-antibiotic control during exposure), meaning that the original sample does not have to be split prior to exposure. Again, the thresholds were defined individually for each antibiotic and pathogen. Using the TTPD_LT_ method, we obtained 100% categorical agreement with gold-standard AST for all antibiotics tested only with *E*. *coli* (Fig 5B) and *K*. *pneumoniae* (Fig 5C) isolates, and with resistant isolates for which the genotypic tests fail to correctly predict the resistance phenotype (red points in Fig 5). When testing *Ebs* (Fig 5D) isolates, we observed 2 errors in which an isolate classified as CRO resistant was called susceptible, resulting in an overall categorical agreement of 88%. Because of these errors, the AUC for *Ebs* isolates tested with CRO was 0.94. Aside from these errors, susceptible and resistant isolates were well separated in all cases, with AUC = 1.000 for all antibiotics tested with *E*. *coli* and *K*. *pneumoniae*. Although we observed 2 errors, using the TTPD_LT_ metric still gave excellent agreement with gold-standard AST and required no splitting of the sample prior to exposure.

To demonstrate one of the major differences between pol-aAST, a phenotypic method, and existing genotypic methods, we challenged the assay with 5 previously characterized isolates that had either (i) no detectable β-lactamase genes or (ii) lacked any genotypic signature predictive of β-lactam resistance. We tested 2 *E*. *coli* and 2 *K*. *pneumoniae* isolates with no detectable β-lactamase genes as measured by both a published genotypic assay designed to screen for 6 β-lactamase gene families [67], as well as the Cepheid Xpert Carba-R test (a commercial, FDA-approved genotypic assay designed to screen for 5 β-lactamase gene families) [68]. These 4 isolates did not test positive in either assay because they lack the genes these assays screen for, despite being resistant (as determined by gold-standard broth microdilution). These 4 tested isolates were resistant to CRO and ETP, and one isolate from each genus was also resistant to MEM. Additionally, we tested a single resistant *Ebs* isolate from the CDC Enterobacteriaceae Carbapenem Breakpoint Panel (AR-Bank #0007). Whole genome sequencing of this isolate (performed by the CDC) revealed no known resistance markers [66], meaning that the mechanism of resistance was uncharacterized. The pol-aAST performed excellently in all cases, and all 5 isolates were correctly categorized as resistant (Figs 4 and 5, red points).

**Fig 6:**
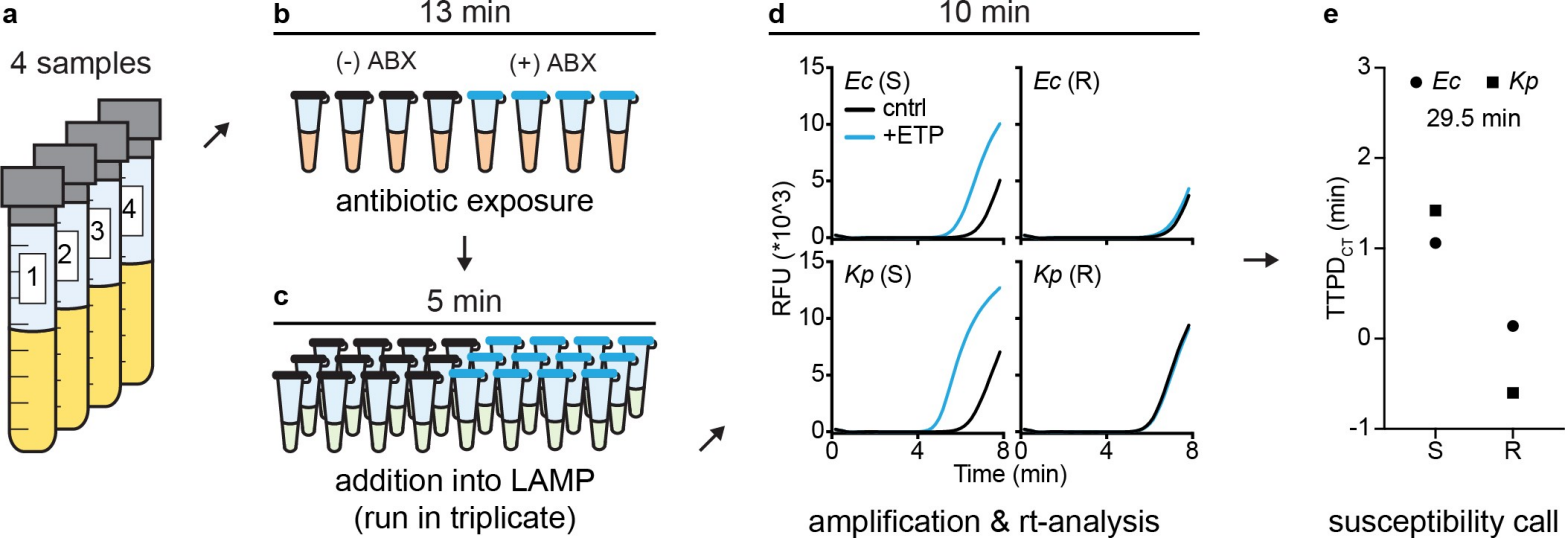
Timed sample-to-answer pol-aAST using contrived urine samples spiked with either *Ec* or *Kp*. (a) Because minimal sample handling is required for pol-aAST, all 4 contrived urine samples were run in parallel. (b) Urine samples were split into control and antibiotic-treated aliquots and incubated at 37°C for 13 min. A timer was started immediately after sample splitting. (c) All samples were added to pre-made LAMP mix and run in technical triplicate. (d) Samples were amplified using LAMP, and the fluorescence of reactions was monitored in real time. Once total fluorescence passed a predetermined threshold (indicating successful amplification), reactions were stopped and TTP values ported into an automated data-analysis spreadsheet. The timer was stopped as soon as the spreadsheet gave susceptibility calls. (e) Comparison of susceptibility calls with gold-standard AST categorization. Total assay time was 29.5 min. Raw data are provided in S3 Table. ABX, antibiotic; AST, antibiotic susceptibility test/testing; cntrl, control; *Ec*, *E*. *coli*; ETP, ertapenem; *Kp*, *K*. *pneumoniae*; LAMP, loop-mediated isothermal amplification; pol-aAST, polymerase-accessibility AST; R, resistant; RFU, relative fluorescent units; rt, real-time; S, susceptible; TTP, time-to-positive; TTPD, time-to-positive difference.

To investigate the sample-to-answer time of the pol-aAST, we performed timed experiments using contrived urine samples (Fig 6). Sample-to-answer time is a critical metric for any assay designed to be used at the POC but is often not reported at all, even for methods claiming to be rapid. In timed experiments, we (i) reduced the exposure time from 15 to 13 min to ensure that all handling could be performed during the 15 min allocated for exposure and (ii) used an automated data-analysis spreadsheet to provide a susceptibility call as soon as the LAMP reactions reached a predetermined threshold (indicating successful amplification). At the initiation of pol-aAST, a timer was started that ran for the duration of the experiment and was stopped once a susceptibility call had been made. The susceptibility of 4 isolates to ETP was tested simultaneously (Fig 6A). The pol-aAST consists of only 3 simple handling steps (Fig 6B–6D), which allowed us to perform pol-aAST in a total time of just 29.5 min, with results in agreement with gold-standard AST (Fig 6E).

**Fig 7:**
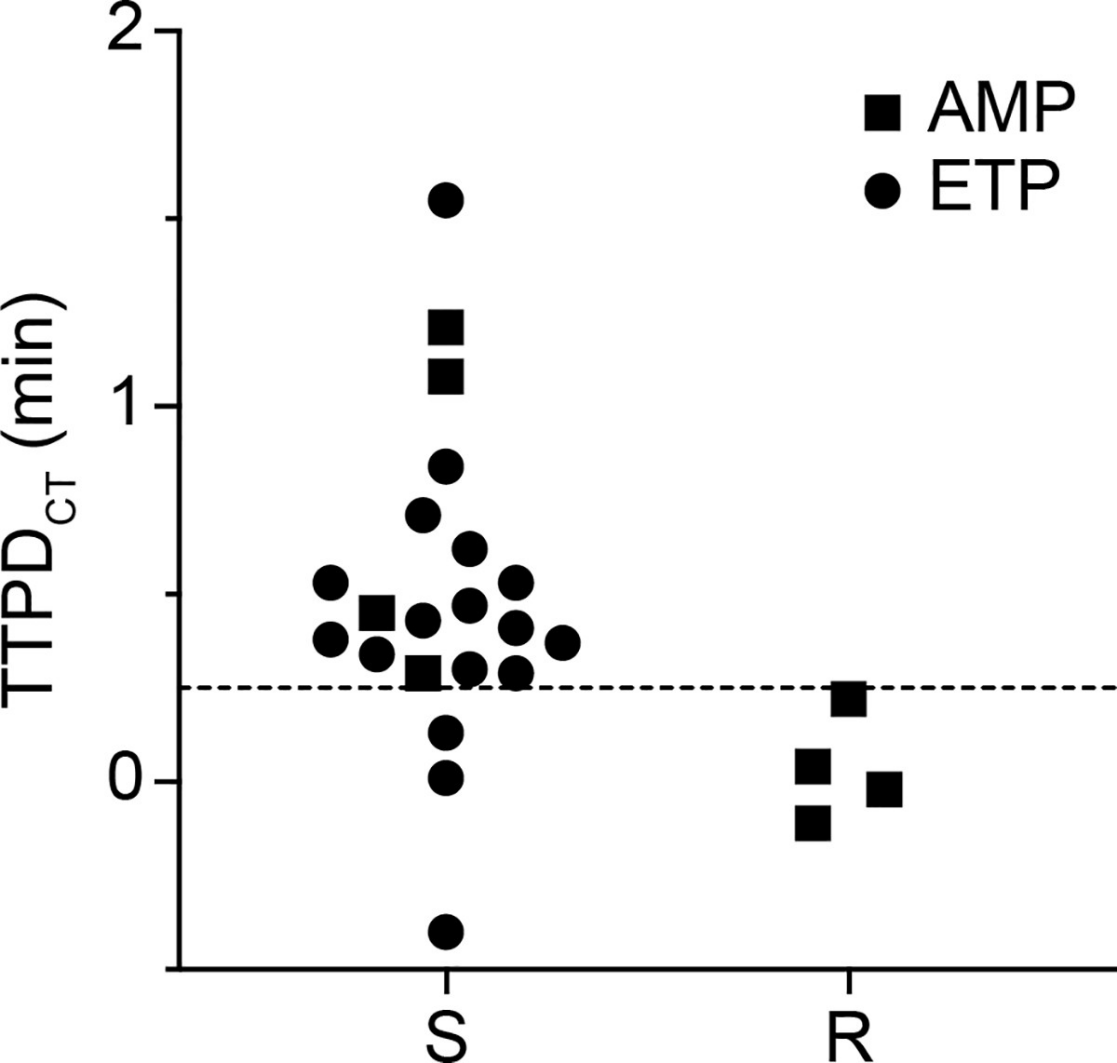
Pilot testing of pol-aAST with clinical UTI samples with a modified protocol (see Methods and Discussion). TTPD_CT_ values for AMP and ETP susceptibility obtained by pol-aAST, with clinical UTI samples containing *E*. *coli*. Each point represents the TTPD_CT_ value for one clinical sample tested once by pol-aAST (S2 and S4 Tables). LAMP was performed in technical triplicate, see S4 Table for values and statistical details. AMP, ampicillin; AST, antibiotic susceptibility testing; ETP, ertapenem; LAMP, loop-mediated isothermal amplification; pol-aAST, polymerase-accessibility AST; R, resistant; S, susceptible; TTPD, time-to-positive difference; TTPD_CT_, TTPD control to treated; UTI, urinary tract infection.

We next ran the pol-aAST on clinical urine samples from patients diagnosed with UTI. These samples were confirmed to be Enterobacteriaceae-positive UTIs by the UCLA CML, and the pol-aASTs were run 3 to 5 d after collection. Initial experiments running the pol-aAST directly on clinical urine samples revealed an insufficient response to antibiotics in some samples. Because we analyzed urine samples that had been stored in a chemical preservative (see **Methods**) for 3 to 5 d after collection, some variation in the response to antibiotics was expected. However, we wished to test whether the delays in the response were indeed due to the phenotypic state of bacteria in these archived samples, and not due to the intrinsic biology of the bacterial strains in these samples. To test, we obtained 25 clinical urine specimens that exhibited an expected heterogeneity, as indicated by the wide range of urinalysis findings (see S2 Table): pH ranged from <5 to 8, specific gravities ranged from <1.005 to >1.060 (above and below the ranges detected in standard urinalysis), and protein, ketone, and bilirubin contents ranged from absent to the maximum measurable by urinalysis. Some samples contained red blood cells, leukocytes, and squamous epithelial cells. Two of the samples were polymicrobial. To ensure a response from bacteria in these specimens, we added a 30-min pre-incubation step of urine with media and increased the duration of antibiotic exposure to 45 min (see **Methods**). We did not optimize these conditions and did not attempt to identify the shortest possible incubation or exposure time. Eight samples were tested for ampicillin (AMP) susceptibility, and 17 samples were tested for ETP susceptibility. Prior to testing clinical samples using AMP, we tested 5 *E*. *coli* isolates using AMP (S1 Fig). Despite the heterogeneity in the urine matrix and the likely nutrient-deprived condition of the bacteria in the urine samples, pol-aAST experiments yielded clean separation between AMP-sensitive and -resistant *E*. *coli*. Additionally, we were able observe a response to ETP in 14 of 17 ETP-sensitive urine samples tested. Overall, we obtained 100% categorical agreement for determination of AMP susceptibility (4/4 susceptible and 4/4 resistant; Fig 7) and observed a response indicating susceptibility to ETP in 14 of 17 (82.4%) confirmed-susceptible samples (Fig 7), including the 2 polymicrobial samples. None of the samples received for testing by the pol-aAST method were ETP-resistant.

## Discussion

The pol-aAST method enables rapid, organism-specific measurement of susceptibility to β-lactams—the most important class of antibiotic for gram-negative infections—thus providing the critically missing piece needed to develop a POC AST for this global health threat. The genera of isolates and the β-lactams used in this proof-of-concept study were intentionally chosen—*E*. *coli*, *K*. *pneumoniae*, and *Ebs*—and are responsible for the majority of CRE infections globally [8,11–13] (in some areas of the US, *K*. *pneumoniae* is responsible for up to 90% of CRE infections [5]). It is for this reason that *E*. *coli*, *K*. *pneumoniae*, and *Ebs* together make up the majority of isolates in the CDC’s Enterobacteriaceae Carbapenem Breakpoint panel, a collection of isolates designed specifically to challenge carbapenem-susceptibility tests in Enterobacteriaceae [66]. CRO, used broadly for a variety of infections because of its broad coverage and tolerability, was chosen as a representative third-generation cephalosporin. Similarly, ETP and MEM were chosen as clinically representative carbapenems [69]. When testing clinical samples, AMP was chosen because of its high resistance prevalence and thus availability of resistant samples (55.8% of clinical urine samples received by the UCLA CML are AMP resistant [70]). We chose ETP as a representative carbapenem.

The pol-aAST has 2 important requirements: (i) amplification conditions that are not fully lytic and (ii) release of NAs only from cells that are susceptible to the β-lactam to which they are exposed. If cells fully lyse, as they do in PCR, there is no difference in amplification between control and treated aliquots in susceptible isolates (Fig 2). It is only under partial-lysis conditions, as in LAMP, that cell integrity is preserved long enough to yield a substantial TTPD. Cell integrity, and rate and degree of lysis, will also depend on the identity of the organism, as well as its growth rate. In partial-lysis conditions, most NAs are still protected inside cells in the control aliquot, whereas a significant portion of NAs are released and start amplifying immediately in the treated aliquot. We know from previous work [60] that the speed of an optimized bulk LAMP reaction makes it is difficult to linearly correlate TTP and NA concentration, unless very sensitive real-time measurements are made. Based on the magnitude of the differences in TTP observed here and the results measuring NA release (Fig 3), we suspect that both the state of NAs (inside intact cells versus inside or outside damaged cells) and the differences in concentration of free NAs contribute to the TTPDs observed. Cell-wall defects and damage are also likely to increase the penetration of amplification reagents into DNA trapped inside the remains of susceptible treated cells especially under the elevated temperature of the amplification reaction. We chose LAMP because we have shown previously that it is a rapid and specific isothermal amplification chemistry [60]. However, other non-lytic isothermal amplification chemistries could also be investigated. Additionally, DNA release (Fig 3) could be measured to determine susceptibility using PCR if combined with a filtration step; we have not evaluated the pros and cons of this approach in this paper. Lastly, alternative or modified accessibility-based AST approaches will likely need to be developed for different organisms, as we have done for *Neisseria gonorrhoeae* [71].

To demonstrate the flexibility of the pol-aAST method and the simplicity of the workflow, we investigated 2 approaches for determining susceptibility. The first, measuring TTPD_CT_, gave 100% categorical agreement and uses a standard antibiotic-exposure step wherein one aliquot serves as the control and the other aliquot is exposed to an antibiotic. The second, measuring TTPD_LT_, differs in that only a single aliquot of the original sample is used during the antibiotic-exposure step. After exposure, this aliquot is compared with a fully lysed control aliquot, which could be extracted at any point during the assay. Using only a single aliquot of the original sample during exposure reduces the challenges of fluid handling and metering, which will be valuable when developing fully integrated devices. When using a control and treated aliquot, both aliquots must have precisely metered volumes, and the heating required during exposure must be performed on both aliquots. Both methods showed excellent categorical agreement with gold-standard broth microdilution, and the choice of approach will be dictated by future device architecture.

To illustrate the value of phenotypic approaches, we evaluated pol-aAST using isolates that tested negative for β-lactamase genes and isolates that lack a predictive genotype (e.g., no β-lactamase production, no modified porins, no modified penicillin-binding proteins), based on published and commercial genotypic assays [67], and CDC classification based on the ResFinder database [72], respectively. The antibiotic susceptibility of isolates lacking β-lactamases cannot be detected by current, FDA-approved genotypic methods, yet bacteria that do not produce β-lactamases can constitute 11% to 71% of CRE infections [4,73,74]. Using pol-aAST, all 5 of these isolates were correctly categorized as resistant.

Sample-to-answer time directly reflects the speed of diagnostics in practice and is a major factor in how likely a diagnostic is to be adopted. In general, the shorter the sample-to-answer time, the more valuable the test is and the more feasible for use at the POC. With urine as the contrived sample matrix, pol-aAST was able to be completed in <30 min. This timescale is on par both with suggested time-frames for rapid POC diagnostics [63,64] and measured times of patient visits [75]. Additionally, because urine involves relatively simple sample-handling steps, we were able to perform 4 ASTs in parallel when testing contrived samples. The ability to run several samples in parallel demonstrates the potential to multiplex multiple antibiotics, which will be important for the next steps, including the design of integrated devices.

We have demonstrated direct testing of 25 clinical UTI samples using the pol-aAST with changes to the workflow (see Methods). However, even with the heterogeneity of clinical urine specimens (see urinalysis in S2 Table), including 2 polymicrobial samples that were correctly classified as ETP-S, the pol-aAST demonstrated good agreement with gold-standard broth dilution. The ability to handle polymicrobial samples was predictable based on the molecular specificity of NA-based methods. We expect this work to set the foundation for future improvements when using clinical samples.

The pol-aAST method demonstrates a rapid NA-based phenotypic AST for β-lactams and CREs. As with any academic report of an innovative diagnostic technology development, this work has limitations in the breadth of its scope and level of technological maturity. The following work would further extend the clinical applicability of this study and will be necessary for translation into a system suitable for regulatory approval and clinical use. First, the pol-aAST needs to be further developed and evaluated with fresh clinical urine samples from patients; here, we have used chemically preserved samples that were 3 to 5 d old, which likely decreased the response time of bacteria to antibiotics. We expect fresh clinical samples to show more rapid and consistent responses; this hypothesis remains to be tested. We note that many state-of-the-art phenotypic AST methods are initially published without validation of performance directly on clinical samples, e.g. a recent breakthrough demonstrating phenotypic AST on isolates and on blood cultures [58]. Urine is a relevant matrix for a CRE diagnostic because UTIs are the most common source of CRE isolates [76], and because of the large number of hospital-acquired infections that involve catheters or other long-term indwelling medical devices [11], where CRE infections cause major problems. Second, to expand the scope of this approach, other sample types such as blood and blood cultures should be tested (in combination with appropriate pathogen-isolation and pathogen-enrichment technologies). Third, only categorical (S/R) agreement with the gold-standard method was tested here. While in the majority of cases a rapid categorical AST is clinically actionable, testing samples with a range of minimum inhibitory concentrations (MICs), including those with intermediate resistance, would further broaden the scope of the method. Fourth, we have not tested pol-aAST against heteroresistant samples. However, these are more common in gram-positive organisms [77] and are not common in gram-negative organisms. Fifth, the pol-aAST chemistry should be integrated with microfluidic devices so the AST can be performed directly on clinical samples with minimal user intervention. Sixth, the performance of these integrated devices will need to be evaluated in preclinical and clinical studies.

We emphasize that the specific pol-aAST described in this paper, just like other innovative rapid ASTs [60,78–81], is not intended to be the sole test to guide treatment. Even though pol-aAST is based on detection of pathogen-specific NAs and can therefore provide pathogen ID, we anticipate that in a clinical workflow pol-aAST would be performed after a separate rapid pathogen ID step [17,18,20]. This ID step would then allow an unambiguous choice of the appropriate rapid AST. Furthermore, pol-aAST would likely be combined with rapid AST for other antibiotics, such as fluoroquinolones that can be used to treat CRE infections. AST methods that rely on similar underlying chemistries are more likely to be successfully integrated together. Isothermal amplification of pathogen-specific NAs appears to be a promising approach for AST, and we have already shown how a rapid fluoroquinolone AST can be performed in 30 min using digital LAMP [60]. Integration of pol-aAST with these complementary methods and translation to a distributable diagnostic will enable (i) improved antibiotic stewardship by reducing empiric use of carbapenems for Enterobacteriaceae, (ii) improved patient outcomes by detecting CRE infections for which carbapenems would be ineffective, and (iii) more cost-effective surveillance of CRE outbreaks.

We envision that exploratory and mechanistic research inspired by pol-aAST will lead to a new generation of AST diagnostics. Additional mechanistic studies, such as those involving visualizing bacterial response to antibiotics [82,83], would clarify the effects of different antibiotics on the responses measured in pol-aAST for different pathogens. To evaluate whether pol-aAST can be broadened beyond CREs and β-lactams, these studies would include organisms with cell envelopes that differ from Enterobacteriaceae (e.g., gram-positives) and other antimicrobials that affect the cell envelope, such as antimicrobial peptides [84] or vancomycin. It would also be desirable to evaluate pol-aAST with more amplification chemistries, including modified LAMP assays [85,86] and other isothermal chemistries [87–89], such as recombinase polymerase amplification (RPA), that are actively being developed and can be performed at lower temperatures. Ultimately, this new generation of AST diagnostics will be integrated with the rapid ID methods being developed [17,18,20] and with future rapid NA-based AST methods for additional antibiotics and pathogens. For example, we have developed the nuclease-accessibility AST (nuc-aAST) [71], which measures accessibility of DNA to nucleases and was used to perform a rapid test of antibiotic susceptibility on the fastidious organism *N*. *gonorrhoeae*. In contrast to the pol-aAST, the nuc-aAST enhances antibiotic-induced damage using surfactants after the antibiotic-exposure step and performs full cell lysis. Ultimately, to address the broad diversity of antibiotic-resistant pathogens, it is clear that integrated, multiplexed POC devices that incorporate multiple rapid phenotypic AST methods are needed. Innovative methods based on antibiotic-induced accessibility of NAs to enzymes are promising for generating such ASTs for multiple antibiotics and pathogens in an approach that is intrinsically compatible with other rapid AST methods [60] and with rapid pathogen ID [17,18,21,22].

## Methods

### Ethics statement

Remnant urine samples from patients with confirmed UTI were received by UCLA CML and released to the Caltech researchers under UCLA IRB #19–001098. The UCLA IRB waived the requirement for informed consent and/or assent and/or parent permission under 45 CFR 46.116(d) for the entire study. No identifying information was obtained by the Caltech team, and the research was determined to be exempt by Caltech IRB (applications #18–0858 and #19–0909).

### Study design

The objective of this study was to develop a rapid phenotypic AST for β-lactams based on DNA accessibility to polymerase for use with Enterobacteriaceae. To calculate the sample size necessary to validate the method (Figs 4 and 5), the Methods and Equation 5 from Banoo and colleagues [90] were used as described previously [60], namely, we suspected that the specificity and sensitivity of the nuc-aAST method would be 95% with a desired margin of error of ±10%. Under these conditions, 18.2 (or 19) samples must be tested with the nuc-aAST method and compared to the gold standard. We performed 36 ASTs with isolates susceptible to the antibiotic being tested and 46 ASTs with isolates resistant to the antibiotic being tested.

### Isolates, growth conditions, and antibiotic exposure conditions

We obtained 25 de-identified clinical isolates from the UCLA CML and the CDC’s Enterobacteriaceae Carbapenem Breakpoint panel [66]. In the case of isolates obtained from the UCLA CML, MICs were determined as described previously [59]. Genotypic testing of the 2 *E*. *coli* and 2 *K*. *pneumoniae* isolates selected for their lack of known β-lactamase genes was performed by UCLA CML using a previously published assay [67] and separately at the Keck School of Medicine of USC using the FDA-approved Cepheid Xpert Carba-R test. Whole genome sequencing of the single *Ebs* isolate selected for its lack of known resistance genes was performed by the CDC [66]. All isolates were stored as glycerol stocks at −80°C. Glycerol stocks were streaked onto Trypticase Soy Agar with 5% sheep’s blood (Becton Dickinson, Franklin Lakes, NJ) and grown overnight at 37°C or resuspended directly in liquid media. Prior to experiments, a small clump of cells was resuspended from plates or glycerol stocks in 2 mL Brain Heart Infusion Broth (BHI; Becton Dickinson) at 37°C + 5% CO_2_ with 500 rpm shaking for 2 to 4 h until visibly turbid. OD_600_ of the cultures was then measured, and working cultures were prepared at an OD_600_ of 0.01–0.07 and grown for 50–145 min at 37°C + 5% CO_2_ with 500 rpm. Working cultures were then diluted 10X into control and treated aliquots for antibiotic exposure. For validation experiments, antibiotic exposure was performed in 100 μL volumes consisting of 80 μL Mueller Hinton II Broth (MHB; Becton Dickinson), 5 μL nuclease-free H_2_O (NF-H_2_O), 5 μL 20X antibiotic stock solution, and 10 μL of working culture. In control aliquots, antibiotic stock solution was replaced with NF-H_2_O. For filtration experiments, antibiotic exposure was performed in 100 μL volumes consisting of 65 μL MHB (Becton Dickinson), 21 μL NF-H_2_O, 4 μL 25X antibiotic stock solution, and 10 μL of working culture. In control aliquots, antibiotic stock solution was replaced with NF-H_2_O.

### Antibiotic stocks

CRO disodium salt hemi(heptahydrate) (Sigma, St. Louis, MO), ETP sodium salt (Research Products International, Prospect, IL), and MEM trihydrate (TCI, Portland, OR) were used to create 1.0 mg/mL antibiotic stock solutions in NF-H_2_O based on manufacturer-reported purity, aliquoted, and stored at −80°C. AMP sodium salt (Sigma, St. Louis, MO) was used to create 10.0 mg/mL antibiotic stock solutions in NF-H_2_O based on manufacturer-reported purity, aliquoted, and stored at −80°C. Aliquots were only thawed and used once on the days of experiments.

### Comparison of amplification methods

In order to compare amplification using LAMP and PCR, *E*. *coli* isolates were exposed to 0.5 μg/mL ETP for 15 min. Samples were then transferred directly into either PCR or LAMP mix on ice. Amplification was started immediately. qPCR was performed on a Roche LightCycler 96 using SsoFast EvaGreen Supermix (BioRad, Hercules, CA); 10 μL reactions were used. 10% of the final reaction volume was template. Published primers targeting the 23S rRNA genes of Enterobacteriaceae were used [91] at a final concentration of 500 nM. Cycling conditions consisted of 3.0 min at 95°C, followed by 35 cycles of 95°C for 10 s, 60°C for 10 s, and 72°C for 15 s. Fluorescence was measured using the SYBR Green channel after each 72°C extension step. LAMP was performed on a BioRad CFX96 using the following conditions: 10 μL reaction volume containing 1X Isothermal Reaction Buffer II (NEB), 5 mM MgSO_4_ (NEB), 1.4 mM dNTPs (NEB), 320 U/mL *Bst* 3.0 (NEB), and 2 μM Syto-9 (Thermo Fisher); 10% of the reaction volume was template. Primer sequences (designed to target the 23S rRNA genes of Enterobacteriaceae) and concentrations have been described previously [60]. Cycling conditions consisted of 2.0 min at 12°C (while lid was heating), followed by 120 cycles of 70°C for 10 s. Fluorescence was measured using the SYBR Green channel every 10 s (after each cycle). We also ran an analogous LAMP reaction in the absence of Tween-20 (which is normally present in Isothermal Reaction Buffer II; NEB), to test for a potential difference in lysis efficiency; however, the resulting reaction rates were substantially lower than when Tween-20 was included.

### Filtration experiments

Filtration experiments were performed using *E*. *coli* isolates exposed to 0.5 μg/mL ETP for 15 min. Immediately following exposure, cultures were passed through 0.22 μm, 1.5 mL cellulose acetate centrifuge tube filters (Corning Costar Spin-X, Corning, NY). DNA retention by the filters was <7% when measured by quantifying purified Lambda phage DNA (NEB) before and after filtration. Quantification was performed using ddPCR (QX200, BioRad). In filtration experiments, 50 μL of sample was added to the filter and centrifuged for 4 min at 1,000 rcf. DNA was extracted from both the feed and filtrate using QuickExtract DNA Extraction Solution (Lucigen, Middleton, WI). Samples were diluted 10X into extraction buffer and extracted according to manufacturer instructions. The concentration of the single copy *E*. *coli uidA* gene was then quantified in the feed and filtrate extractions. The percentage of *E*. *coli* DNA in the filtrate was calculated as the filtrate concentration divided by the feed concentration. ddPCR was performed using QX200 ddPCR Supermix for EvaGreen (BioRad); 10% of the final reaction volume was template. Published primers targeting the *uid*A gene in *E*. *coli* were used [92] at a final concentration of 500 nM. Cycling conditions consisted of 5.0 min at 95°C, followed by 40 cycles of 95°C for 30 s, 60°C for 30 s, and 72°C for 30 s, with final dye stabilization steps of 4°C for 5.0 min followed by 90°C for 5.0 min.

### pol-aAST validation with clinical isolates

For pol-aAST validation experiments, *E*. *coli* and *Enterobacter* spp. isolates were exposed to either 2.0 μg/mL CRO, 0.5 μg/mL ETP, or 1.0 μg/mL MEM. *K*. *pneumoniae* isolates were exposed to either 2.0 μg/mL CRO, 1.0 μg/mL ETP, or 1.0 μg/mL MEM. Some isolates were run multiple times on different days. If this was the case, the average TTPD_CT_ and TTPD_LT_ are reported for that isolate. All isolates were exposed to antibiotics for 15 min in 100 μL reaction volumes in 200 μL PCR tube strips. After 15 min of antibiotic exposure, 10 μL of samples were transferred as template to LAMP reaction mix (as described earlier) on ice in technical triplicate. Amplification was immediately started.

### Timed sample-to-answer using contrived urine samples

Timed sample-to-answer experiments were performed in the same fashion as pol-aAST validation experiments, except with the following modifications. Following initial growth and measurement of OD, isolates were resuspended in fresh, never-frozen, pooled human urine from healthy donors (Lee BioSciences). Additionally, a timer was started as soon as samples were added to the antibiotic exposure conditions. *E*. *coli* and *K*. *pneumoniae* isolates were exposed to 0.5 and 1.0 μg/mL ETP (respectively) for 13 min. The duration of 13 min was chosen to ensure that all handling steps could be completed within the first 15 min of the assay. Amplification was performed until all reactions reached a fluorescence value of 1,000 relative fluorescent units (RFU) or greater. Amplification was then stopped, and TTP values were copied into a spreadsheet pre-populated with formulas to automatically output susceptibility calls. The timer was stopped once a susceptibility call had been determined.

### Testing of pol-aAST with clinical samples

UCLA CML performed urinalysis, confirmation of UTI, pathogen isolation and ID, and subsequent gold-standard AST using broth microdilution. Gold-standard AST results were sent to Caltech researchers on the same day samples were received. Enterobacteriaceae-positive samples were shipped at ambient temperature to Caltech in BD Vacutainer Plus C&S preservative tubes (Becton Dickinson, Catalog Number 364951) containing a boric acid preservative. The pol-aAST experiments were performed directly on these samples within 3–5 d of their collection at UCLA. Urine samples were first warmed up to 37°C without shaking for 30 min, to approximate temperature of freshly collected urine. Then, 30 μL of urine was diluted into 70 μL of Cation-adjusted MHB (BD) containing 0.1% Tween-20 (Teknova, Hollister, CA) and placed at 37°C with shaking at 750 rpm for 3 min. Samples were then centrifuged at 5,000 rcf for 2 min. The supernatant was removed, and the sample was resuspended in 100 μL of MHB. Samples were then incubated for 30 min at 37°C with 750 rpm shaking. Antibiotic exposure was performed in a final volume of 100 μL, after transfer of 20 μL of incubated sample to 80 μL of the exposure condition: 75 μL of MHB and 5 μL of 20X antibiotic stock solution in NF-H2O for treated aliquots, or 75 μL of MHB with 5 μL of NF-H2O alone for control aliquots. For measurement of ETP susceptibility, the exposure condition contained a final concentration of 1μg/mL of ETP. Aliquots were incubated at 37°C with shaking for 20 min. For measurement of AMP susceptibility, the antibiotic-exposure condition contained a final concentration of 16 μg/mL of AMP, and aliquots were incubated at 37°C with shaking for 45 min. The control and treated aliquots were subjected to a set of dilutions to account for variable bacterial load of the samples and resolution within the working range of the LAMP reaction. Following dilution, 1 μL of the control and treated aliquots was added to each LAMP reaction well. There were 3 technical replicates (3 LAMP reaction wells) for each condition (control and treated). We measured the TTP for the reactions at each dilution, and then selected the dilution that yielded a control TTP value later than 4.7 min. The TTP results from this dilution were used to calculate TTPD_CT_ (and determine susceptibility). Samples with a TTPD_CT_ > 0.25 min were considered susceptible, while samples with TTPD_CT_ ≤ to 0.25 min were considered resistant. The susceptibility determination of the pol-aAST method was then compared to the gold-standard culture results obtained by the UCLA CML to measure assay performance.

### Statistical analysis

Significance referenced in the text for Fig 2 were calculated using GraphPad Prism 8.0 software from an unpaired, two-tailed *t* test comparing the averages of 3 replicate Cq values of each control sample to each treated sample. A significance value of 0.02 was used for statistical significance. All percent release values (Fig 3) and TTPD values (Figs 4–6) were calculated using Microsoft Excel. Data were plotted using GraphPad Prism 8.0 software. Thresholds for determining susceptibility in TTPD_CT_ and TTPD_LT_ plots were set halfway between the lowest S and highest R values for each organism/antibiotic combination. For preliminary tests with clinical samples, we defined a TTPD_CT_ of above 0.25 min for a susceptible determination; this value would be further defined in a subsequent larger-scale clinical trial.

## Supporting information

S1 Table Clinical isolates used in this study. Isolates were obtained from the UCLA CML and the CDC’s Enterobacteriaceae Carbapenem Breakpoint panel. The MIC of each isolate (based on broth microdilution performed by UCLA CML) are provided.
(XLSX)

S2 Table Clinical urine samples from patients with UTIs used in this study. Clinical samples were obtained from the UCLA CML. MICs based on broth microdilution performed by UCLA CML are provided along with urinalysis results.
(XLSX)

S3 Table Raw data and calculated error for all pol-aASTs performed using clinical isolates. Cqs and TTPs are provided.
(XLSX)

S4 Table Raw data and calculated error for all pol-aASTs performed using clinical UTI samples. Cqs and TTPs are provided.
(XLSX)

S5 Table Raw data for amplification curves shown in Fig 2. Technical triplicate values are provided for control and treated (0.50 μg/mL ETP) samples run using LAMP and PCR. Grey lines in Fig 2 represent standard deviation of the triplicate samples calculated using Graphpad Prism.
(CSV)

S6 Table Raw data for percentage of DNA release shown in Fig 3. Negative percentage release values were set to zero before averaging. Averages and standard deviations of each isolate/antibiotic combination were calculated using GraphPad Prism.
(XLSX)

S1 Fig Validation of pol-aAST TTPD_CT_ method using AMP. *E*. *coli* isolates were exposed to 16 μg/mL AMP for 15 min. Threshold was set halfway between the lowest susceptible and highest resistant TTPD_CT_ value. Data are in S3 Table. R, resistant; S, susceptible.
(TIF)

S1 Text Detailed statement of author contributions. (PDF)

## Abbreviations

AMP: ampicillin
AST: antibiotic susceptibility test/testing
AUC: area under the curve
BHI: Brain Heart Infusion Broth
CDC: Centers for Disease Control and Prevention
CML: Clinical Microbiology Laboratory
Cq: quantitation cycle
CRE: carbapenem-resistant Enterobacteriaceae
CRO: ceftriaxone
ddPCR: droplet digital PCR
ETP: ertapenem
FDA: Food and Drug Administration
ID: identification
LAMP: loop-mediated isothermal amplification
MEM: meropenem
MHB: Mueller Hinton II Broth
MIC: minimum inhibitory concentration
NA: nucleic acid
NEB: New England Biolabs
NF-H2O: nuclease-free H2O
nuc-aAST: nuclease-accessibility AST
PCR: polymerase chain reaction
POC: point of care
pol-aAST: polymerase-accessibility AST
qPCR: quantitative PCR
RFU: relative fluorescent unit
ROC: receiver operating characteristic
RPA: recombinase polymerase amplification
TTP: time-to-positive
TTPD: time-to-positive difference
TTPD_CT_, TTPD: control to treated
TTPD_LT_, TTPD: lysed-control to treated
UTI: urinary tract infection
WHO: World Health Organization
