# Supplementary figures and images for: Differential DNA accessibility to polymerase enables 30-minute phenotypic β-lactam antibiotic susceptibility testing of carbapenem-resistant Enterobacteriaceae
Source: PLoS Biol. 2020 Mar 19;18(3):e3000652. doi: 10.1371/journal.pbio.3000652 (PMC7081982; doi:10.1371/journal.pbio.3000652)

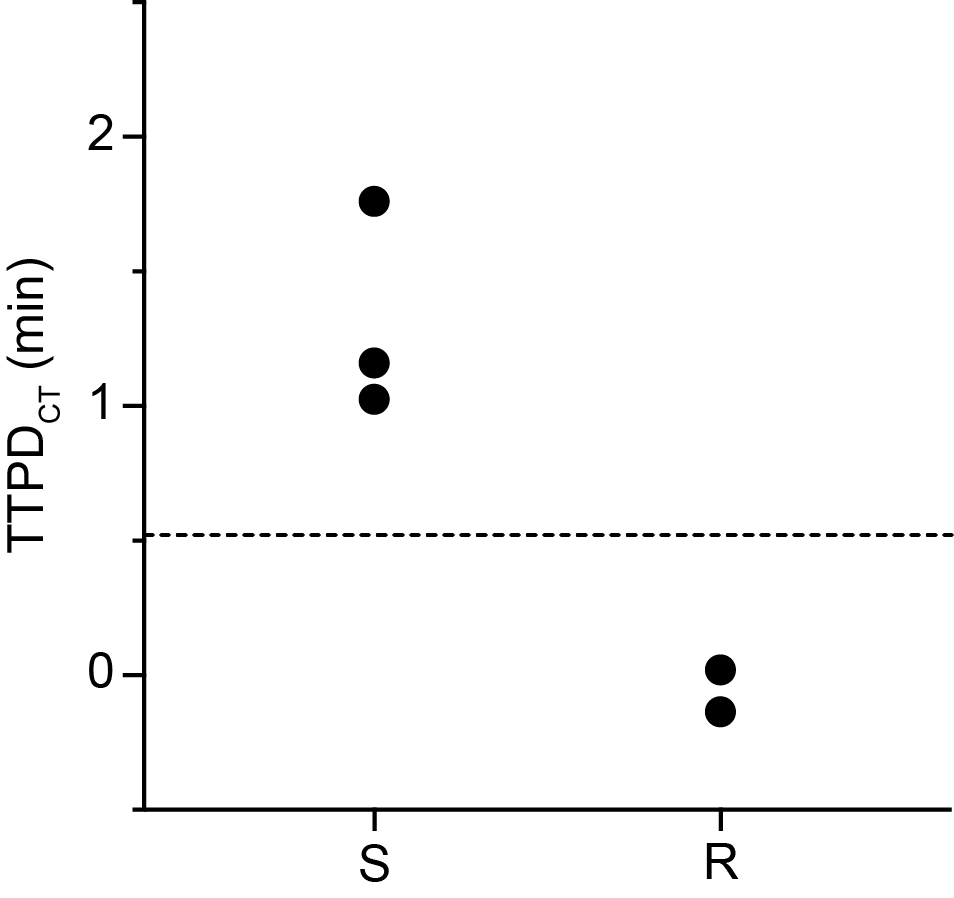

Supplement: S1 Fig — E. coli isolates were exposed to 16 μg/mL AMP for 15 min. Threshold was set halfway between the lowest susceptible and highest resistant TTPDCT value. Data are in S3 Table. R, resistant; S, susceptible. (TIF) [file pbio.3000652.s007.tif]
