# Supplementary material for: Differential DNA accessibility to polymerase enables 30-minute phenotypic β-lactam antibiotic susceptibility testing of carbapenem-resistant Enterobacteriaceae
Source: PLoS Biol. 2020 Mar 19;18(3):e3000652. doi: 10.1371/journal.pbio.3000652 (PMC7081982; doi:10.1371/journal.pbio.3000652)
Supplement: S1 Text — (PDF) [file pbio.3000652.s008.pdf]

Supplemental for:

Differential DNA Accessibility to Polymerase Enables 30-minute Phenotypic β-lactam Antibiotic Susceptibility Testing of Carbapenem-resistant Enterobacteriaceae

Nathan G. Schoepp[1], Eric J. Liaw[2], Alexander Winnett[2], Emily S. Savela[2], Omai B. Garner[3], Rustem F. Ismagilov[1,2]*

[1]Division of Chemistry and Chemical Engineering, California Institute of Technology

[2]Division of Biology and Biological Engineering, California Institute of Technology
1200 E. California Blvd., Pasadena, CA, United States

[3] Department of Pathology and Laboratory Medicine, UCLA, Los Angeles, CA

*Author to whom correspondence should be addressed

Detailed author contribution statement

NGS, EJL, AW, ESS, and RFI contributed to conceiving the method, revising the manuscript, and interpretation of experimental results. NGS developed the sample handling workflow and performed all experiments for comparison of amplification methods, validation, and timed sample-to-answer experiments. NGS was the major contributor to manuscript preparation and prepared all figures. EJL performed filtration experiments, reviewed relevant medical literature, and contributed to manuscript writing. NGS and AW tested clinical samples using the modified workflow. ESS performed early experimental work to link beta-lactam exposure to differential nucleic acid readout, analyzed data from validation experiments, and developed TTPD metrics. OBG provided clinical guidance on the selection of clinical isolates and clinical samples and coordinated and provided oversight of clinical-sample collection at UCLA, including technical assistance to UCLA staff. RFI supervised and guided the project, and helped compose the manuscript.
